# Research on Processing Temperature of Atmospheric Pressure Microwave Plasma Based on Fused Silica Etching

**DOI:** 10.3390/mi17070771

**Published:** 2026-06-25

**Authors:** Xiang Wu, Bin Fan, Qiang Xin, Dawei Luo, Bo Gao, Wei Li, Zhentian Guan, Qiang Chen

**Affiliations:** 1 Institute of Optics and Electronics, Chinese Academy of Sciences, Chengdu 610209, China; 2 University of Chinese Academy of Sciences, Beijing 100049, China

**Keywords:** optical processing, plasma, etching behavior, temperature characteristics, processing method

## Abstract

This study investigates the processing temperature characteristics and etching behavior of fused silica using an atmospheric pressure microwave plasma jet. The temperature distribution within the processing region was measured in real time via infrared thermography. The effects of microwave input power, argon flow rate, and CF_4_ flow rate on the processing temperature were systematically examined using a single-factor approach. Experimental results reveal a strong positive correlation between the plasma temperature and microwave power. The temperature initially rises and then declines with increasing argon flow, peaking at 3 slm, while it increases and eventually stabilizes with higher CF_4_ flow. Fixed-point etching demonstrates that the etching rate increases with rising processing temperature. Furthermore, heat accumulation during prolonged dwell time leads to a nonlinear increase in the removal rate. This effect can be effectively mitigated by employing a multi-segment processing strategy, enabling more stable and controllable material removal. The effectiveness of this processing method has also been verified on a fused quartz sub-mirror.

## 1. Introduction

Fused silica (SiO_2_) is an extremely popular material for optical components. Known for its high transmittance, excellent thermal and chemical stability, and good mechanical properties, it is an ideal material for ultra-precision optical elements. Currently, ultra-precision machining technologies for fused silica optics primarily include computer-controlled small tool polishing [1], bonnet polishing [2], magnetorheological finishing (MRF) [3], and ion beam figuring (IBF) [4]. Among these techniques, contact-based methods represented by small tool polishing and bonnet polishing offer high machining efficiency. However, the machining process of optical components is often accompanied by contact stress [5,6], which inevitably induces surface or subsurface damage, thereby reducing the laser-induced damage threshold of the optics [7]. This is also highly detrimental for machining thin mirrors. Furthermore, rigid polishing tools lack the flexibility to rapidly adapt to changes in the curvature of aspheric components. Magnetorheological finishing provides excellent removal characteristics and high controllability, but the properties of the magnetorheological fluid are susceptible to contamination from the external environment. Ion beam figuring offers high machining accuracy but requires vacuum conditions, has an extremely low material removal rate, and involves high costs; thus, it is typically applied only in the final processing stage. With the growing demand for low-damage, high-performance machining of optical components, non-contact atmospheric pressure plasma processing methods are attracting increasing attention.

Plasma-based optical machining is a non-contact material removal method that operates in an atmospheric environment. It involves exciting reactive gases such as CF_4_ or SF_6_ to form a plasma jet containing active species, which then chemically reacts with silicon-based components like fused silica to achieve material removal. Researchers from the Leibniz Institute of Surface Modification in Germany conducted studies on plasma jet machining for optical components used in X-ray synchrotron radiation and space satellite systems. They achieved material volume removal rates of 40 mm^3^/min for SiO_2_ and 50 mm^3^/min for ULE optical component [8]. Cranfield University in the UK successfully reduced the form error of a 100 mm aperture SiO_2_ spherical optical component and a 400 mm aperture ULE optical component to 16 nm RMS and 30 nm RMS, respectively, using Reactive Atom Plasma Technology [9,10], demonstrating the engineering application potential of plasma machining technology for high-efficiency, damage-free manufacturing of optical elements. By adjusting discharge parameters and leveraging the high radical density and non-contact processing characteristics of plasma, Zhang et al. utilized atmospheric pressure inductively coupled plasma to achieve rapid exposure and detection of the subsurface damage layer in SiC [11], validating the high-efficiency removal capability of plasma for machining-induced damage layers. Li et al. conducted research on atmospheric plasma machining technology, achieving a surface roughness of 2.39 nm RMS on quartz optical components and successfully fabricating freeform continuous phase plates [12]. Xin et al. proposed an inductively coupled atmospheric pressure plasma process and investigated the surface morphology evolution of polished fused silica [13,14]. Jiao et al. simulated and experimentally verified the evolution behavior of the microscopic material removal profile during atmospheric pressure jet plasma processing using the level-set method [15]. Jin et al. studied the material removal mechanism and deposition suppression methods in atmospheric pressure inductively coupled plasma (ICP) [16]. Yu et al. investigated the aerodynamic characteristics of atmospheric pressure plasma jets based on computational fluid dynamics to support the optimization of inductively coupled plasma torch design [17].

To date, atmospheric pressure plasma processing methods have achieved remarkable research results in enhancing material removal efficiency, eliminating machining-induced damage, and correcting form errors. Atmospheric pressure plasma etching has been widely adopted for processing silicon-based materials. Beyond the aspects mentioned above, numerous factors in the atmospheric pressure plasma machining process—such as power, gas flow rate, and processing time—directly influence the temperature within the plasma processing zone, thereby altering the actual material etching rate. Some scholars have investigated the temperature variation characteristics of inductively coupled plasmas and capacitively coupled plasmas [18,19]. However, research specifically on the temperature within the atmospheric pressure microwave plasma processing zone and its actual impact on the material removal of fused silica has not been published. Therefore, this paper conducts the following research based on atmospheric pressure microwave plasma: Firstly, we measured the temperature distribution characteristics of the plasma processing zone using a thermal imager. Employing a single-factor method, we investigated the influence of microwave input power, the flow rates of the excitation gas (Ar), and the reactive gas (CF_4_) on the temperature within the plasma processing zone, thereby identifying an effective process window for low-temperature processing. Subsequently, we analyzed the influence mechanism of the plasma processing zone temperature on the removal rate. Fixed-point etching experiments on fused silica optical components were conducted to study the effects of dwell time and processing mode on the actual etching rate. Furthermore, by examining the dynamic changes in the plasma processing zone temperature, we investigated the nonlinear increase between the etching rate and the processing dwell time caused by heat accumulation in the processing zone. Finally, the effectiveness of atmospheric pressure microwave plasma low-temperature shaping was verified on a fused quartz optical component. This work lays a foundation for subsequent research on ultra-precision optical machining processes using atmospheric pressure microwave plasma.

## 2. Materials and Methods

The atmospheric pressure microwave plasma processing system is primarily composed of a plasma generation device, a mechanical motion control system, a gas supply system, and an external module. The plasma generation device includes a solid-state microwave source and a plasma generator. The mechanical motion control system features three motion stages (X, Y, and Z axes) and a numerical control system capable of controlling the precise movement of the plasma generation device. The gas supply system provides regulated inputs of three gases: Ar, CF_4_, and O_2_. Among these, the rare gas Ar, which is readily excited to generate plasma, serves as the excitation gas. The fluorine-containing gas CF_4_ acts as the reactive gas and is the primary source for the plasma-induced reactive etching of the fused silica component. A small amount of O_2_ is primarily used to suppress the formation of etch deposits [20]. The flow rates of Ar, CF_4_, and O_2_ are controlled by commercially available mass flow controllers (Beijing Qixing Huachuang Electron Co., Ltd., Beijing, China). The external module is an infrared thermal imager, used to measure the temperature within the processing region on the component surface where the plasma acts. Under the ionization excitation from the microwave source, reactive species F* generated within the plasma will react with the surface of the fused silica optical component. This reaction converts the material at the surface layer of the solid substrate into volatile gaseous compounds through the plasma etching process. For the silicon-based material fused silica, the etching reaction can be expressed as follows [19]:

SiO_2_ + CF_4_ → SiF_4_↑ + CO_2_↑ (1)

**Figure 1:**
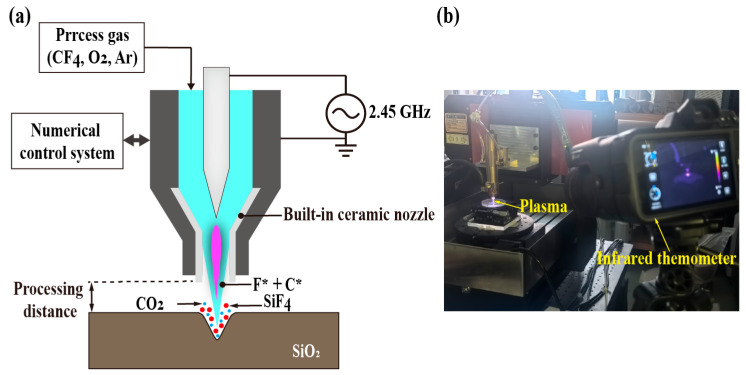
Atmospheric pressure microwave plasma processing system: (**a**) schematic diagram; (**b**) physical picture.

As the plasma etching reaction proceeds continuously, material removal occurs in the processing region where the substrate surface interacts with the plasma, ultimately enabling the processing of the fused silica optical component by the plasma. Figure 1 shows a schematic view of the plasma generator, which was installed on a computer numerical control (CNC) x-y-z-table. In the experiment, a built-in ceramic nozzle with an inner diameter of 2 mm was used. A physical picture of the processing system is shown in Figure 1.

**Figure 2:**
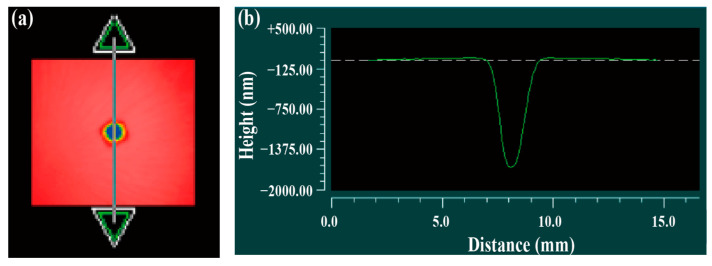
Material removal distribution: (**a**) material removal profile; (**b**) cross-sectional profile.

To facilitate the investigation and comparison of the influence of the atmospheric pressure microwave plasma reaction zone temperature on the material removal rate of fused silica, the material removal profiles from single-point plasma etching under different conditions were measured. The material removal function profile of the atmospheric pressure jet plasma is typically near-Gaussian, which is similar to traditional non-contact ion beam machining. Figure 2 shows the material removal profile and the corresponding central cross-sectional profile of a fixed-point etch pit generated by the atmospheric pressure microwave plasma with a dwell time of 10 s. This etch was performed under the following parameters: microwave input power of 60 W, nozzle distance of 5 mm, Ar flow rate of 2.3 slm, O_2_ flow rate of 10 sccm, and CF_4_ flow rate of 5 sccm, as measured by an interferometer (GPI150 by Zygo, Middlefield, CT, USA). Evidently, the PV value (peak removal depth) of the material removal profile can serve as a parameter for comparing the actual etching rates.

## 3. Temperature Measurement Method

**Figure 3:**
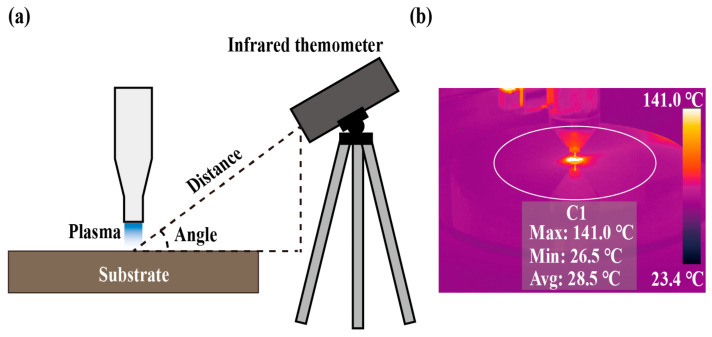
Temperature measurement of the processing area: (**a**) Schematic diagram of the infrared thermometer; (**b**) on-site measurement view.

In the field of manufacturing, common methods for measuring the temperature of a processing zone include the thermocouple method [21], probe method [22], spectral intensity method [23], and infrared thermography [24]. The characteristics of plasma, which contains positively and negatively charged particles, are highly susceptible to disturbance from external metallic objects. Furthermore, plasma probe methods are unsuitable for atmospheric pressure environments. Consequently, non-contact methods such as the spectral intensity method and infrared thermography are prioritized. Most commercially available spectrometers typically measure the relative intensity of different emission spectra within the processing zone rather than absolute intensities, making it difficult to determine the plasma processing zone temperature based on spectral emission theory. In contrast, the technologically mature infrared thermography method enables convenient, rapid, and reliable measurement of the temperature field within the plasma processing zone. Therefore, as shown in Figure 3, infrared thermography was employed in this study to measure the temperature in the plasma processing zone. To ensure measurement accuracy and the reliability of experimental results, the following principles were adhered to during actual measurement: (1) A suitable measurement position was selected, with the measurement distance and angle of the infrared thermal imager fixed. (2) The ambient temperature was maintained constant, and the material emissivity was set to match that of the actual workpiece substrate material. During the experiment, the measurement distance and angle of the infrared thermography were set to approximately 55 mm and 34°, respectively, and the emissivity of the workpiece substrate material was 0.91.

**Figure 4:**
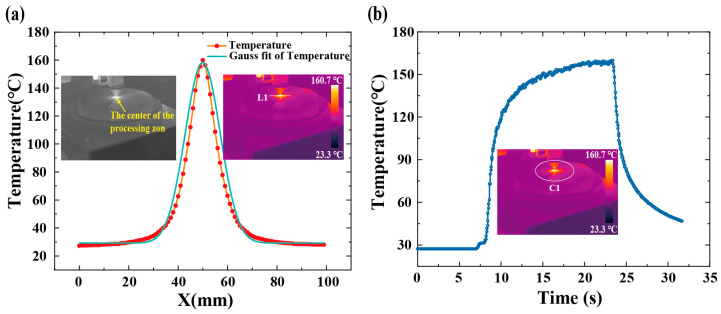
The temperature of the component surface during plasma processing: (**a**) cross-sectional temperature distribution of the processing zone and (**b**) the dynamic change in the temperature at the center over processing time.

Figure 4 shows the cross-sectional temperature distribution in the processing zone of the fused silica optical component measured by the infrared thermal imager (Wuhan Gould IR Technology Co., Ltd., Wuhan, China) and the dynamic change in the temperature at the center over processing time, respectively. The process parameters were as follows: microwave input power of 30 W, Ar flow rate of 3 slm, O_2_ flow rate of 5 sccm, CF_4_ flow rate of 35 sccm, nozzle distance of 5 mm, and a single-point processing time of 15 s. It can be clearly observed that the temperature is highest at the center of the plasma processing zone and decreases towards the edges, with the cross-sectional temperature distribution profile approximating a Gaussian shape. From Figure 4, it is evident that after the plasma jet begins to move over the processing area, the temperature in the processing zone first increases rapidly and then rises gradually as the etching time prolongs. When the processing concludes and the plasma jet moves away from the component surface, the temperature in the processing zone drops swiftly initially, followed by a gradual decrease. In this study, to analyze the influence of processing parameters on the temperature within the plasma processing zone, the temperatures measured in subsequent experiments are all the maximum temperature values recorded at the center of the plasma processing zone under different processing conditions.

## 4. Results and Discussion

### 4.1. Influence of Processing Parameters on Plasma Processing Temperature

#### 4.1.1. Effect of Microwave Input Power

In an atmospheric pressure microwave plasma processing system, the microwave input power is a core control parameter, as it directly determines the level of energy injected into the system. The magnitude of the input power not only influences whether the plasma can be successfully ignited and maintained stably but, more crucially, it governs the power density of the plasma jet, the concentration of active species, and the overall thermal state, thereby ultimately determining its processing capability.

When investigating the influence of different processing parameters on the temperature of the plasma processing zone, the processing distance (5 mm) and the dwell time (15 s) were set as fixed values. The fused silica substrate employed in the experiment has a diameter of 60 mm and a thickness of 7 mm. In this experiment, to avoid phenomena such as plasma ignition failure, arcing, a weak jet, or instability, the discharge parameters for different microwave input power levels tested in this section are listed in Table 1.

**Figure 5:**
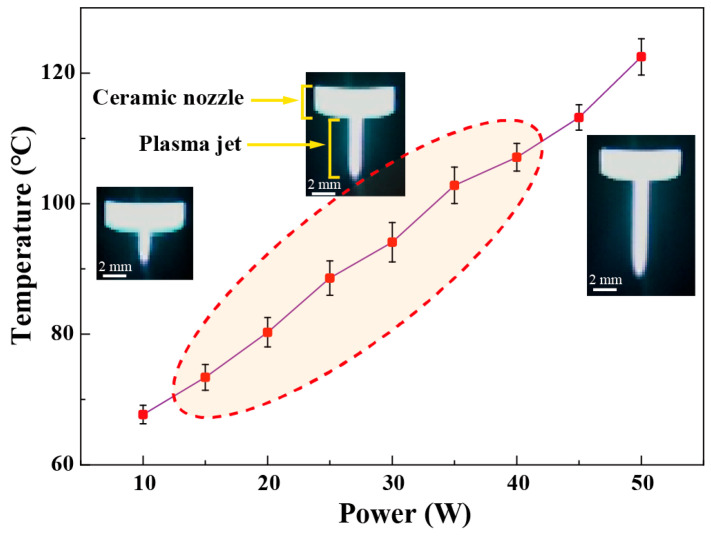
Effect of microwave input power on the temperature of the plasma processing zone.

Figure 5 shows the variation curve of the plasma processing zone temperature with microwave input power. Evidently, the temperature in the plasma processing zone increases significantly with rising input power. This is primarily attributed to the fact that higher power increases the microwave electric field intensity, allowing more microwave energy to be absorbed efficiently by free electrons. These high-energy electrons subsequently transfer their kinetic energy into the internal and thermal energy of neutral gas atoms/molecules through frequent collisions, leading to a rise in gas temperature and dissociating more active etching species, which enhances the etching rate. Concurrently, it was observed experimentally that as the power increased, the plasma jet morphology exhibited volumetric expansion, with both its length and diameter increasing, which aligns with the aforementioned theory. Furthermore, when the power exceeded 40 W, the temperature in the processing zone began to rise at an accelerated rate. Therefore, to ensure a relatively low plasma processing temperature, the microwave power in practical processing should be controlled below 40 W.

#### 4.1.2. Effect of Argon Flow Rate

In atmospheric pressure microwave plasma processing systems, the selection of the working gas and its flow rate parameters decisively influence the plasma characteristics. Argon was chosen as the primary working gas in this experiment due to its low ionization energy and abundance of metastable energy levels. These properties make it easy to ignite and sustain in the microwave field, and it acts as an excellent energy carrier and reaction medium by efficiently transferring energy to subsequently introduced reactive gases via the Penning effect. As shown in Table 2, this section details the experimental parameters set for different argon flow rates, systematically investigating the governing effect of argon flow rate on the temperature within the plasma processing zone.

**Figure 6:**
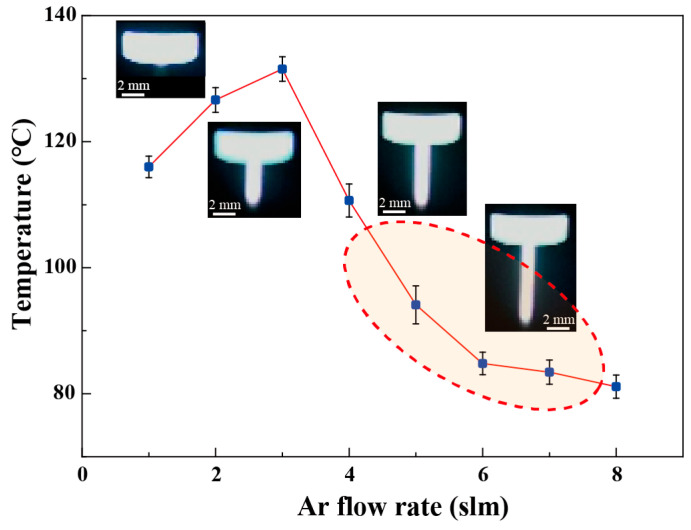
Effect of Ar flow rate on the temperature of the plasma processing zone.

The experimental results, depicted in Figure 6, reveal a non-monotonic relationship: the temperature of the plasma jet processing zone initially increases with rising argon flow rate, reaches a maximum, then decreases, eventually tending to level off. This phenomenon unveils the complex dynamic balance between energy injection and dissipation within the system. In the low flow rate regime, with constant microwave input power, a lower gas flow rate implies that the gas molecules per unit volume receive a higher energy density, leading to an increased power density. Simultaneously, the convective cooling effect of the lower-velocity gas on the plasma core region is weaker, resulting in energy accumulation within the zone and manifesting as a steady rise in jet temperature. As the argon flow rate exceeds 3 slm, the temperature curve inflects and begins to decline. At this stage, the residence time of gas molecules within the microwave energy absorption region is shorter, reducing the energy coupling efficiency. Furthermore, enhanced forced convective cooling causes the temperature to gradually decrease from its peak. Upon entering the high flow rate regime, the temperature exhibits a gentler declining trend. In this state, intense convective cooling becomes the dominant mechanism. The high-velocity gas flow physically stretches the plasma jet, intensifying its mixing with the surrounding ambient air and dissipating additional energy. Consequently, the input energy is primarily consumed to maintain the ionization state of the plasma rather than heating the gas, leading to the plateau in the temperature decline curve. Experiments also observed that high flow rates can physically stretch and compress the plasma jet, making it thinner and longer, and potentially causing instability (flickering or breaking). Therefore, the argon flow rate should generally be neither too low nor too high.

#### 4.1.3. Effect of Carbon Tetrafluoride Flow Rate

The introduction of carbon tetrafluoride, a typical fluorine-containing reactive gas, into the plasma serves not only to provide active fluorine radicals for functional purposes like surface etching but also significantly alters the fundamental physical properties of the plasma, particularly its energy balance and thermodynamic state, as its flow rate changes. To analyze in detail the influence of the carbon tetrafluoride flow rate on the processing zone temperature, the CF_4_ flow rate was gradually increased from 0 to 75 sccm according to the discharge parameters outlined in Table 3.

**Figure 7:**
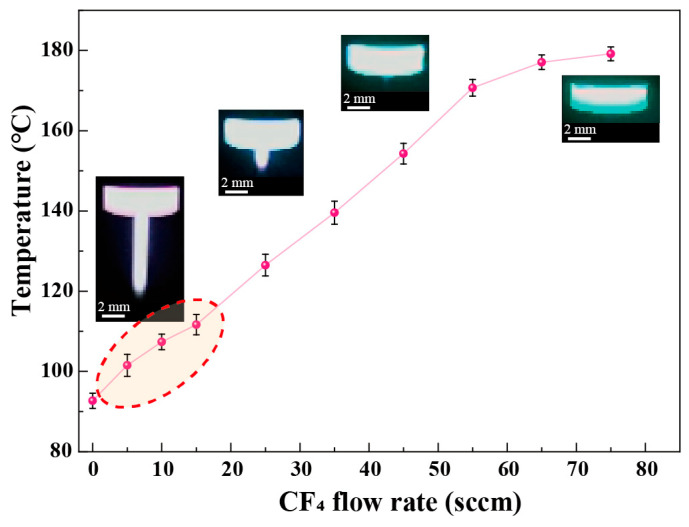
Effect of CF_4_ flow rate on the processing zone temperature.

The relationship between the plasma processing zone temperature and the CF_4_ flow rate is shown in Figure 7. Experimental observations indicate that the temperature exhibits a distinct two-stage characteristic with increasing CF_4_ flow: the temperature rises rapidly as the CF_4_ flow increases from 0 to 55 sccm; beyond 55 sccm, the temperature increase slows and gradually saturates. CF_4_ molecules possess high bond energy, and their dissociation is a strongly endothermic process, requiring the absorption of substantial energy from high-energy electrons and metastable argon atoms (Ar*). This process efficiently converts electron kinetic energy into gas thermal energy through vibration-translation relaxation. Furthermore, compared to monatomic Ar, polyatomic CF_4_ molecules possess more vibrational and rotational degrees of freedom, significantly enhancing the system’s ability to convert electrical energy into thermal energy. Consequently, during the initial stage, the processing zone temperature increases rapidly with the rising CF_4_ flow rate. When the CF_4_ flow rate becomes excessively high, it overly depletes the high-energy electron density and average energy, leading to a decrease in electron temperature, which in turn weakens the ability to sustain efficient ionization and energy transfer. Although the total input power remains constant, the energy allocation for gas heating reaches its upper limit. Therefore, once the CF_4_ flow rate exceeds the critical value (55 sccm), the plateau observed in the temperature curve in Figure 7 signifies the dynamic saturation of energy distribution. Simultaneously, the increase in CF_4_ flow rate is extremely low relative to the argon flow rate, resulting in a comparatively weak cooling trend on the plasma from the high CF_4_ flow itself. Furthermore, experiments revealed that increasing the CF_4_ flow rate consumes more energy for molecular dissociation, while the higher molecular weight and enhanced turbulent mixing reduce jet momentum and weaken thermal expansion, leading to faster quenching and shortening of the plasma jet. Therefore, to maintain a lower processing temperature while ensuring a suitable material removal spot size, the CF_4_ flow rate should not exceed 15 sccm.

### 4.2. Study on the Removal Rate by Microwave Processing

#### 4.2.1. Effect of Plasma Processing Temperature on Removal Rate

Unlike large-area capacitive coupled plasma etching conducted in vacuum chambers [25], no strong sheath bias forms on the fused silica substrate surface during atmospheric pressure microwave plasma jet processing. Therefore, the material etching rate at a point (*x*, *y*) for atmospheric pressure microwave plasma can be expressed as [26]:

(2) $$v_{sp}\left(x,y\right)=\frac{n_{r}\left(x,y\right)\cdot Y_{sp}\cdot A_{m}}{\left(\frac{1}{k_{cr}}+\frac{1}{\beta }\right)\cdot N_{A}\cdot \rho_{m}}$$

where $n_{r}\left(x,y\right)$ is the concentration distribution of the chemically active species within the reaction volume; $Y_{sp}$ is the spontaneous etching coefficient of the processed material, representing the number of active chemical species required to remove one atom of the material; $A_{m}$ is the atomic mass of the processed material; $N_{A}$ is Avogadro’s constant; $\rho_{m}$ is the mass density of the processed material; β is the mass transfer coefficient of the chemically active species; $k_{cr}$ is the temperature-dependent etching reaction rate constant. Since the atomic density of the processed material can be expressed as:

(3) $$\rho_{a}=\rho_{m}\cdot \frac{N_{A}}{A_{m}}$$

The spontaneous etching rate $v_{sp}\left(x,y\right)$ can be simplified to:

(4) $$v_{sp}\left(x,y\right)=n_{r}\left(x,y\right)\cdot \frac{Y_{sp}}{\left(\frac{1}{k_{cr}}+\frac{1}{\beta }\right)\cdot \rho_{a}}$$

This section does not delve into whether the etching primarily occurs in the kinetic regime or the diffusion regime. Evidently, when the plasma nozzle is fixed, the process parameters are constant, and the atmospheric microwave plasma discharge is stable, the etching reaction rate constant $k_{cr}$, which is related to the plasma processing zone temperature, directly influences the etching rate. According to the Arrhenius equation, the relationship between the reaction rate constant $k_{cr}$ and the absolute temperature *T* is exponential [27,28]:

(5) $$k_{cr}=A\cdot e^{-\frac{Ea}{RT}}$$

Here, in the context of etching, the reaction rate constant $k_{cr}$ directly determines the magnitude of the etching rate; *A* is the pre-exponential factor, representing the frequency and spatial orientation of reactant molecular collisions, which can be approximately considered constant for a specific reaction; the activation energy *Ea* is the energy barrier that must be overcome for the reaction to occur, an intrinsic property of the material with units of J/mol; *R* is the ideal gas constant; and *T* is the absolute temperature of the material surface in Kelvin. Equations (3) and (4) indicate that an increase in the processing zone temperature causes the value of the exponential term $e^{-\frac{Ea}{RT}}$ to increase, consequently leading to a sharp rise in the reaction rate constant *k* and thus an increase in the etching rate.

#### 4.2.2. Influence of Dwell Time and Processing Mode on Removal Rate

Previous investigations have established the influence of process parameters (e.g., power, gas flow rates) on regulating the bulk plasma characteristics and the temperature of the processing zone, as well as the theoretical basis for the effect of this processing zone temperature on the etching rate. However, under fixed energy and gas inputs, the processing dwell time becomes another critical dynamic factor affecting the final etching outcome. As the plasma jet acts continuously on the material surface, its energy is inevitably deposited into the processing zone via heat conduction and radiation, inducing an evolution of the substrate’s temperature field. This dynamically changing surface temperature can significantly alter the kinetics of surface chemical reactions and the transport efficiency of active species, potentially causing the material removal rate to deviate from an ideal linear model. This section aims to systematically study the influence of dwell time and processing strategy on the material removal function and to reveal the underlying thermal accumulation mechanism.

To investigate the relationship between dwell time and the material removal depth in atmospheric pressure microwave plasma processing, this section designed a comparison between single-cycle and multi-cycle processing modes. Experiments were conducted under fixed process parameters (microwave power: 20 W; Ar/CF_4_/O_2_ flow rates: 3 slm/3 sccm/5 sccm; nozzle distance: 5 mm). Fixed-point etching was performed, and the material removal profile and depth were measured using an interferometer. The experimental parameters for different dwell times are listed in Table 4. Processing points with a single continuous dwell time are recorded as single-cycle processing points, such as points A_1_ to F_1_. Without altering other process parameters, the same five processing points (points A to F) are set for multi-cycle processing, where their total dwell time is divided into increments of 25 s. For example, the processing point (point F) with a dwell time of 25 × 6 s in Table 4 indicates that this point is processed 6 times, each for 25 s. Sufficient time intervals are maintained between each processing cycle to minimize temperature accumulation on the substrate surface.

**Figure 8:**
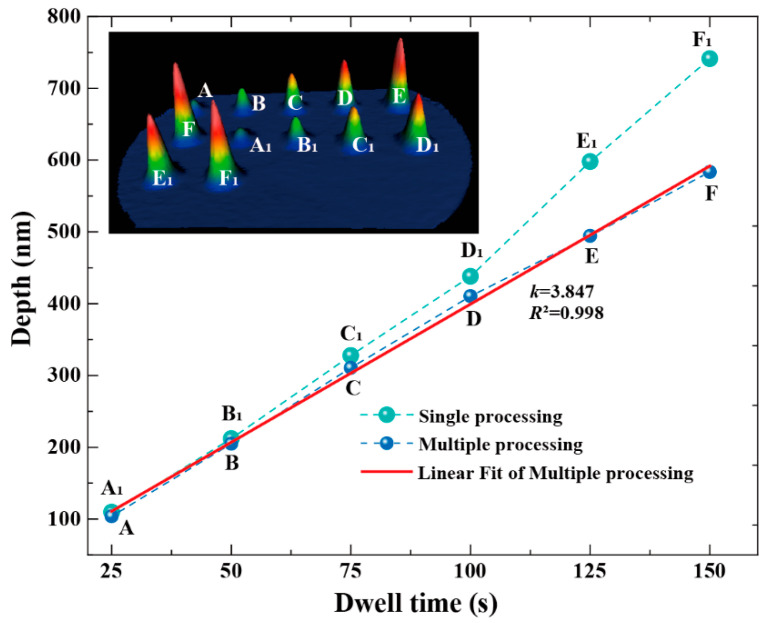
Effect of dwell time on material removal depth under different processing modes.

Figure 8 shows the experimental results of the effect of different dwell times on the material removal depth under the two processing modes. The results indicate that the processing mode significantly affects the relationship between removal depth and dwell time. In the multi-cycle processing mode, the material etch depth exhibited a strong linear relationship with the total dwell time (*R*^2^ > 0.99). This suggests that when thermal accumulation in the substrate is effectively controlled, the etching process stabilizes, resulting in a constant material removal per unit time, consistent with an ideal plasma etching model. In contrast, the single-cycle processing mode, while showing an overall increase in depth with dwell time, displayed a clear non-linear growth trend. Analysis of the experimental data revealed a significant increase in the etching rate as the dwell time prolonged. This deviation from linearity indicates the presence of a dynamically enhancing factor during continuous processing that persistently influences the plasma etching efficiency.

#### 4.2.3. The Central Role of Thermal Accumulation in the Processing Zone

**Figure 9:**
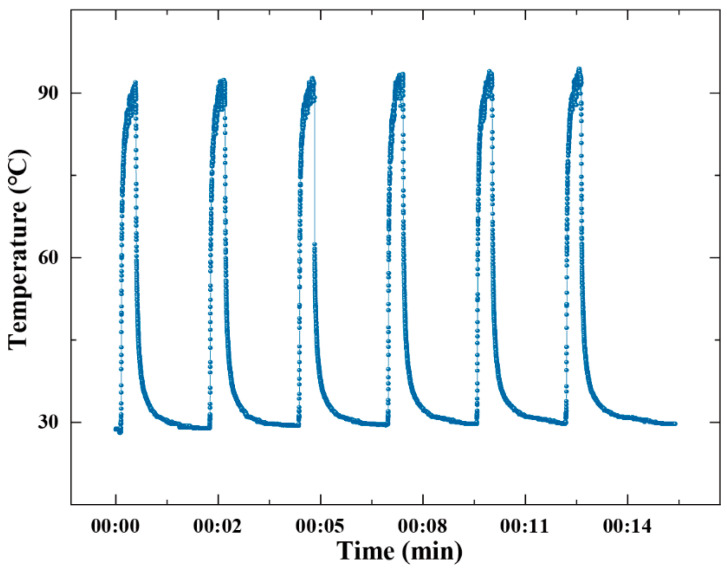
Dynamic temperature evolution in the processing zone under the multi-cycle processing mode.

To elucidate the physical mechanism underlying the aforementioned non-linear phenomenon, an infrared thermal imager was employed to monitor the dynamic temperature changes within the atmospheric microwave plasma processing zone. Figure 9 displays the dynamic temperature evolution in the processing zone for a total dwell time of 150 s, corresponding to the multi-cycle processing mode described in the previous section (6 identical cycles, single-cycle processing time of 25 s, with sufficient 120 s cooling intervals between cycles). The experimental results show that the temperature profile in the multi-cycle mode exhibits highly consistent periodic fluctuations. This indicates that each processing cycle initiates from a similar substrate temperature, effectively resetting the thermal state. This perfectly explains the linear relationship between removal depth and time observed in this mode, as the multi-cycle approach mitigates the time-varying interference of thermal accumulation on plasma etching.

**Figure 10:**
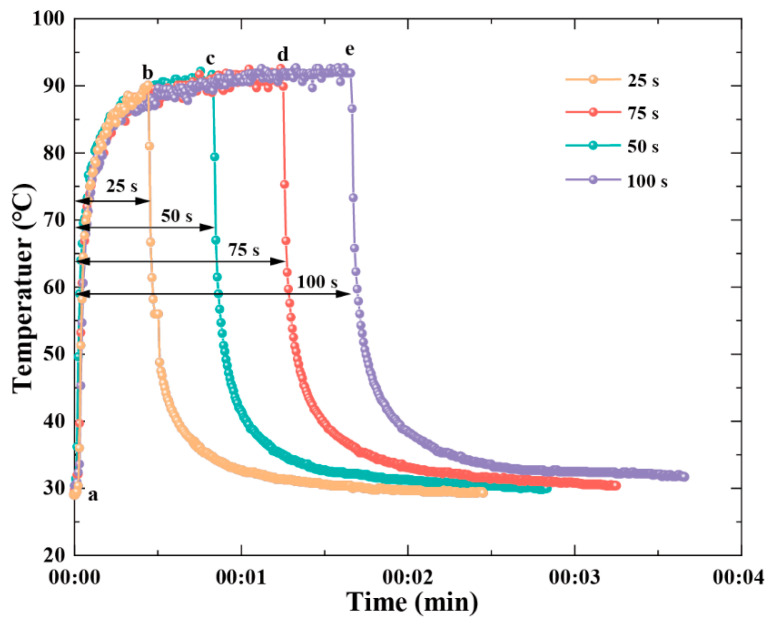
Variation curves of processing temperature under different dwell times.

Figure 10 shows the dynamic temperature changes in the processing zone for single-cycle processing with dwell times ranging from 25 s to 100 s. The results reveal that as processing proceeds, the surface temperature of the fused silica substrate initially increases rapidly due to continuous energy injection from the plasma, followed by a gradual, steady rise. When the continuous processing dwell time is extended, the initial and average temperatures of subsequent extended processing segments are higher than those of the preceding segments. For instance, the initial and average temperatures of segment bc are higher than those of segment ab, and similarly, those of segment cd are higher than those of segment bc. Consequently, in contrast, the temperature curve under the single-cycle processing mode reveals a continuous thermal accumulation effect during atmospheric pressure microwave plasma processing. The elevated surface temperature enhances the plasma etching kinetics through two primary mechanisms: firstly, it directly accelerates the rate of chemical processes, such as the surface fluorination reaction, consistent with the previously mentioned Arrhenius law; secondly, it potentially alters the boundary layer conditions between the plasma and the surface, affecting the transport and adsorption behaviors of active fluorine radicals. These factors collectively lead to a gradual increase in the atmospheric pressure microwave plasma etching rate with prolonged dwell time, manifesting experimentally as the non-linear increase in material removal depth with extended processing dwell time. This non-linear effect between etching rate and processing dwell time, caused by heat accumulation, can be effectively suppressed and mitigated by employing a suitable low-temperature process window for atmospheric pressure microwave plasma processing and adopting a multi-cycle, segmented processing strategy.

### 4.3. Verification of Microwave Plasma Figure Correction Processing

**Figure 11:**
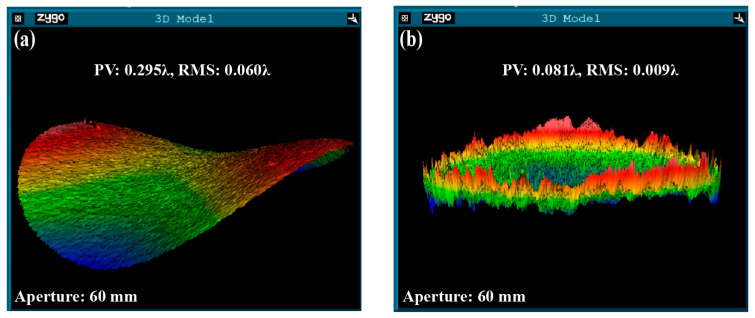
Experimental results of microwave plasma processing: (**a**) initial surface figure error; (**b**) surface figure error after processing.

In Section 4.1, the influence of different discharge process parameters on the temperature of the plasma processing zone was investigated. By fully considering both suitable plasma jet length and low processing temperature, an effective process window for atmospheric pressure microwave plasma low-temperature processing was identified, corresponding to the process parameters indicated by the red dashed lines in Figure 5, Figure 6 and Figure 7. To verify the effectiveness of practically applying atmospheric-pressure microwave plasma for low-temperature surface figure correction, a plasma figure correction experiment was conducted on a 60 mm aperture planar fused quartz sub-mirror. In the experiment, the fixed low-temperature process parameters from Section 4.2.2 were used to obtain the material removal function of the microwave plasma. Furthermore, two cycles of iterative processing were applied to the initial surface figure error of the fused quartz sub-mirror to further reduce the thermal accumulation effect during plasma processing. The experimental results of the microwave plasma surface figure correction are presented in Figure 11.

As can be seen from Figure 11, the initial surface figure error of the fused quartz sub-mirror was RMS 0.060*λ* (*λ* = 632.8 nm). After atmospheric-pressure microwave plasma figure correction processing, the surface figure accuracy converged to RMS 0.009*λ*, resulting in a processing convergence rate of 85%. This result sufficiently demonstrates the effectiveness of using atmospheric-pressure microwave plasma for the low-temperature figure correction processing of optical components. Furthermore, this method exhibits high processing accuracy and holds significant application potential in the ultra-precision machining of optical components.

## 5. Conclusions

This study systematically investigated the influence mechanisms of key process parameters on the temperature within the processing zone and the material removal behavior during atmospheric pressure microwave plasma jet machining of fused silica. Through real-time monitoring via infrared thermography combined with fixed-point etching experiments, the regulatory effects of microwave input power, argon flow rate, and CF_4_ flow rate on the thermodynamic state of the plasma were clarified. Building upon this, the pivotal role of temperature in etching kinetics was analyzed. The main conclusions of this paper are as follows:

(1) The regulatory mechanisms of microwave power, argon, and CF_4_ flow rates on the processing temperature were elucidated. The results indicate that the temperature in the processing zone is positively correlated with microwave power, while argon and CF_4_ flow rates exhibit non-monotonic temperature regulation characteristics by influencing energy coupling, convective cooling, and chemical reaction thermal effects. Based on these findings, a stable process window conducive to low-temperature processing (e.g., power < 40 W, CF_4_ flow rate < 15 sccm) was established. This provides a fundamental process basis for suppressing thermally induced damage and ensuring machining stability.
(2) The central role of the heat accumulation effect during processing was revealed, and the effectiveness of a multi-cycle processing strategy in linearizing the removal process was validated. The research demonstrates that continuous dwell time leads to progressive heating of the substrate, which significantly accelerates the etching reaction via the Arrhenius effect, resulting in a nonlinear increase between removal depth and dwell time. Adopting a segmented processing strategy with sufficient cooling intervals effectively resets the thermal state, restoring a linear and controllable etching process, thereby enhancing the determinism and precision of plasma-based material removal. The effectiveness of high-precision low-temperature figure correction processing using microwave plasma was experimentally verified on a fused quartz sub-mirror. This research not only deepens the understanding of the machining mechanisms underlying atmospheric pressure microwave plasma jets but also provides important theoretical foundations and practical guidance for the high-precision, low-damage ultra-precision machining of optical components, particularly those sensitive to thermal effects. In future work, our primary research focus will be to analyze the interrelationships among various process parameters and to develop an optimized dwell-time scheduling algorithm for compensating thermal errors in plasma machining.

## Figures and Tables

**Table 1:** Discharge parameters under different microwave input power levels.

Parameter	Value
Ar flow rate/slm	5
O_2_ flow rate/sccm	5
CF_4_ flow rate/sccm	4
Input power/W	10, 15, 20, 25, 30, 35, 40, 45, 50

**Table 2:** Discharge parameters under different argon flow rates.

Parameter	Value
Ar flow rate/slm	1, 2, 3, 4, 5, 6, 7, 8
O_2_ flow rate/sccm	5
CF_4_ flow rate/sccm	4
Input power/W	30

**Table 3:** Discharge parameters under different carbon tetrafluoride flow rates.

Parameter	Value
Ar flow rate/slm	5
O_2_ flow rate/sccm	5
CF_4_ flow rate/sccm	0, 5, 10, 15, 25, 35, 45, 55, 65, 75
Input power/W	30

**Table 4:** Experimental parameters for different dwell times.

Atmospheric Pressure Microwave Etching Process	Dwell Time/s
Single processing (points A_1_ to F_1_)	25, 50, 75, 100, 125, 150
Multiple processing (points A to F)	25 × 1, 25 × 2, 25 × 3, 25 × 4, 25 × 5, 25 × 6

## Data Availability

The data are available within the article.
